# Fuel for the road – sugar transport and pollen tube growth

**DOI:** 10.1093/jxb/erw113

**Published:** 2016-03-23

**Authors:** Anke Reinders

**Affiliations:** Department of Plant Biology, University of Minnesota, 140 Gortner Labs, 1479 Gortner Ave., St Paul, MN 55108, USA

**Keywords:** Arabidopsis, glucose signaling, hexokinase 1, hexose transport, major facilitator superfamily, pollen tube growth

**Pollen tubes require the activity of carbohydrate uptake transporters to sustain their high growth rate. The work of Rottmann and colleagues in this issue (pages 2387–2399) provides evidence that expression of a hexose transporter in pollen tubes may be regulated via the hexokinase signaling pathway.**

Pollen, the male gametophyte (haploid generation) of flowering plants, is responsible for delivering sperm cells to the egg sac, a process essential for sexual reproduction. Interaction between pollen and stigma (pollination) initiates secretion in the stigma and pollen germination. The pollen tube emerges and grows through the style to the ovaries where it delivers the sperm cells to the ovule to accomplish fertilization (Box 1) (Edlund *et al.*, 2004). In Arabidopsis, the male gametophyte consists of three cells – one vegetative cell that encloses two sperm cells. Despite this seemingly simple system, about half of all Arabidopsis genes are expressed in pollen (Bock *et al.*, 2006). There has been much recent progress in the field of pollen biology, especially in the area of pollen tube guidance and reception by the female gametophyte (reviewed by Kanaoka and Higashiyama, 2015).

Pollen tubes have been studied intensively as a model system for tip growth, a process vital in diverse cell types from fungal hyphae to cancer cells (Sanati and Geitmann, 2013). Basic research on pollen biology also has many direct practical applications. The vast majority of the world’s harvest depends upon successful plant reproduction. In many crop plants the male gametophyte is sensitive to environmental stress, such as heat and drought, which can lead to serious crop losses (Zinn *et al.*, 2010). Understanding pollen function is therefore also of important practical interest.

## Carbohydrate uptake into pollen tubes

Pollen tubes can sustain very rapid growth rates (5.3 µm min^–1^ in Arabidopsis; Wilhelmi and Preuss, 1996) and achieve lengths of 25cm or more in maize. Carbohydrates and other storage compounds stored in mature pollen are sufficient to support pollen survival and germination but pollen tubes use carbohydrate secretions from the stylar canal to support growth (Labarca and Loewus, 1973). Since the pollen tube is symplastically isolated from the surrounding tissue of the style and transmitting tract, the activity of membrane transporters is required in order to import nutrients from the sporophyte tissue to contribute to cell wall synthesis and respiration of the growing pollen tube. It is therefore interesting to note that genes encoding membrane transporters are overrepresented in the pollen transcriptome (Bock *et al.*, 2006). Pollen can germinate *in vitro* in relatively simple media, which made the first pollen transcriptome studies possible. More recent work using a semi-*in vivo* method where pollen is applied to a stigma, after which the style is cut – allowing the pollen tubes to grow out onto the medium – showed that certain genes are specifically induced after growth through the pistil tissue (Qin *et al.*, 2009). Interestingly, there are not many examples of sugar transporters for which a function in pollen has been demonstrated. One example is the Arabidopsis sucrose transporter AtSUC1, which can be localized to the plasma membrane in pollen tubes. Loss of function affects pollen germination *in vitro* and causes segregation distortion *in vivo* (Sivitz *et al.*, 2008).

However, pollen tubes also express a cell wall-localized invertase (Singh and Knox, 1984) and are capable of hydrolysing all of the sucrose in germination media to fructose and glucose (Ylstra *et al.*, 1998). It is not known to what extent pollen tubes take up sucrose compared to its hydrolysis products fructose and glucose. There are a large number of hexose transporters expressed in pollen and direct evidence for their involvement in pollen function has probably been hampered by redundancy. In Arabidopsis there are 14 members of the SUGAR TRANSPORT PROTEIN (STP) family alone, and six of them are expressed in pollen (reviewed in Büttner, 2010; Rottmann *et al.*, 2016). *STP2*, *STP4*, *STP6*, *STP9* and *STP11* are expressed at different stages of pollen development. *STP4*, *STP10* and *STP11* are expressed in pollen tubes and may be necessary for the uptake of hexoses from the surrounding tissue. Even though all evidence points to a role for STPs in pollen, no pollen-related loss-of-function phenotype has been described for any of the STPs.

## Glucose repression of *STP10* via hexokinase 1

The paper by Rottmann *et al.* (2016) describes the characterization of Arabidopsis STP10. Two aspects are especially interesting for understanding the role of STPs in pollen: evidence for possible hexokinase-dependent repression of expression and for expression pattern regulation by intragenic domains. Expression of *STP10* in yeast was used to show that it encodes a hexose transporter with high affinity for glucose. STP10 also transports galactose and mannose, but not fructose. While no impairment of pollen function was found in an *stp10* loss-of-function mutant, possibly due to redundancy, Rottmann *et al.* provide intriguing insight into the regulation of *STP* expression in pollen. *STP10* is highly induced during pollen germination, both *in vitro* and even more in semi-*in vivo* pollen tubes (Qin *et al.*, 2009; Rottmann *et al.*, 2016). The authors show that expression is only detectable after pollen germination and that the protein is localized to the pollen tube plasma membrane. Induction was lost during *in vitro* germination of pollen when 50mM glucose was added to the medium; fructose and mannose did not have this effect, nor did adding 50mM glucose and 50mM fructose at the same time. Expression of *STP4*, another pollen tube-expressed hexose transporter, was affected in the same way. The downregulation of expression of *STP10* and *STP4* no longer occurred in two independent hexokinase 1 mutants. Hexokinase 1 has a dual role, both as a glucose sensor and as an enzyme that converts glucose to glucose-6-phosphate (Sheen, 2014). The findings by Rottmann *et al.* suggest that the glucose signal that leads to the decrease in *STP10* and *STP4* expression may be transmitted via the hexokinase pathway.

**Box 1. Pollen tube growth**

**Figure:**
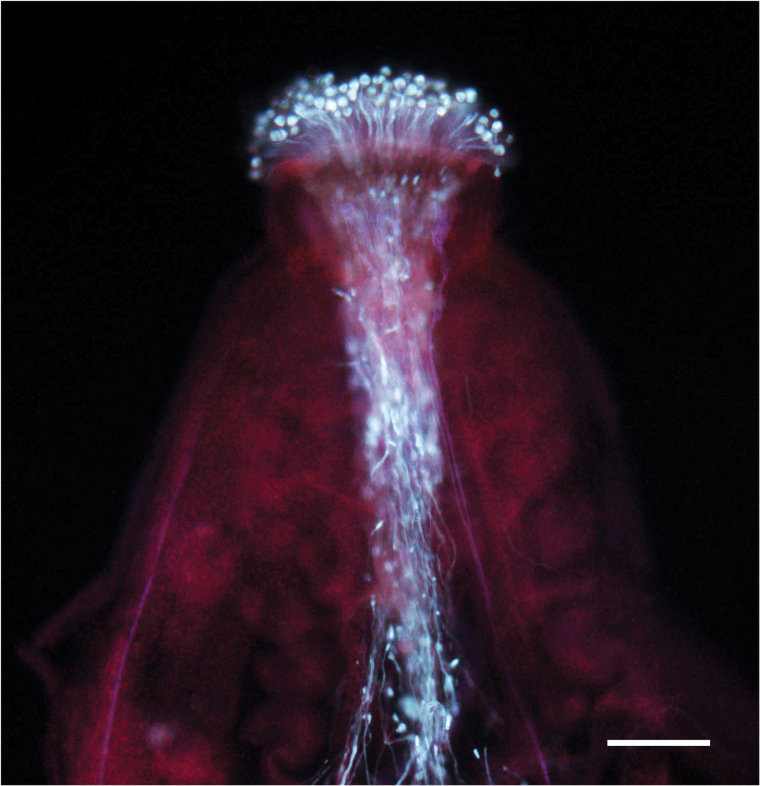

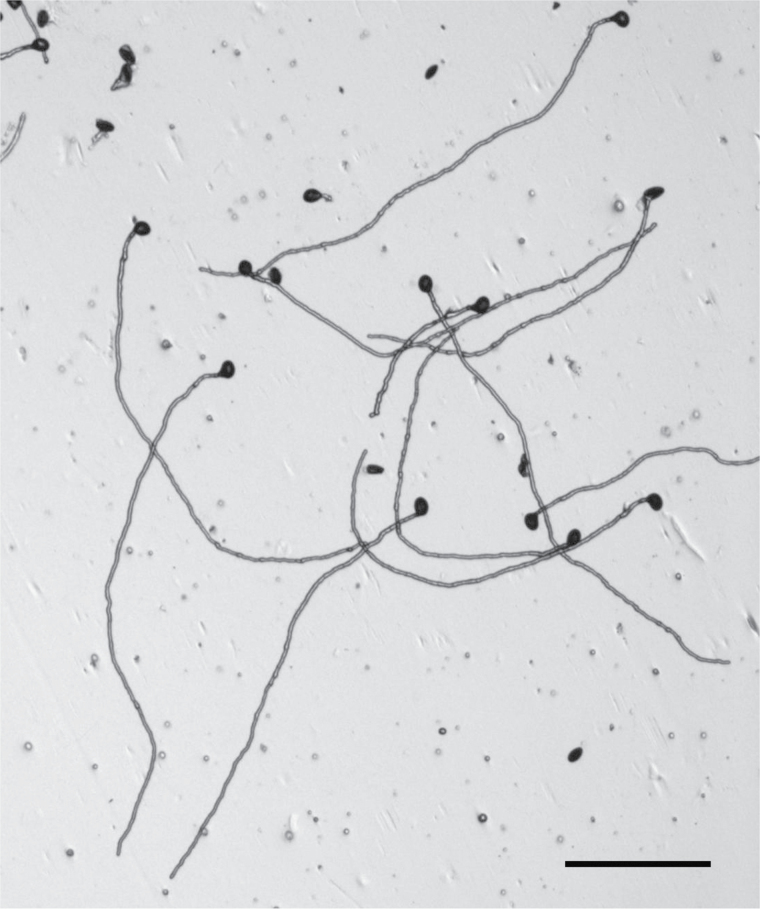
The images show pollen tubes growing through the ovary to reach the egg cells in wild-type Arabidopsis (above, scale bar = 50 µm; aniline blue staining) and *stp10-1* pollen tubes *in vitro* (below, scale bar = 100 µm). Courtesy of Dr Ruth Stadler.

More information is available concerning transcriptional control of *STP10*. In a triple *myb97 myb101 myb120* mutant, the pollen tubes fail to burst upon reaching the synergid and therefore do not deliver the sperm to the ovule. Interestingly, in this mutant a number of genes are no longer induced during pollen germination, among them *STP10* (Leydon *et al.*, 2013). *STP10* may therefore be a direct or indirect target of MYB regulation. The three *MYBs* themselves are induced during growth through the pistil tissue.

An additional interesting finding by Rottmann *et al.* (2016) is that intragenic regions of *STP10* seem to be involved in providing specificity to the expression of *STP10*. A promoter–GUS fusion showed expression in many more tissues than a whole-gene–GUS fusion, indicating that a transcriptional repressor may be present in intron, exon or UTR sequences.

## Next steps for understanding sugar uptake by pollen

Future work will undoubtedly have to address the issue of redundancy, and the creation and analysis of double or higher-order multiple mutants in several hexose/sugar transporter genes. We also need to know what sugars are available in the transmitting tract, especially in Arabidopsis. Such data would provide a realistic picture of the conditions encountered by growing pollen tubes and help us understand how they get the nutrition necessary for rapid growth.
